# Impact of High-Dose Cefepime During the Initial 48 h on Intensive Care Unit Survival in Sepsis: A Retrospective Observational Study

**DOI:** 10.3390/antibiotics15010088

**Published:** 2026-01-15

**Authors:** Tsukasa Kuwana, Kosaku Kinoshita, Yuma Kanai, Yurina Yamaya, Ken Takahashi, Satoshi Ishizuka, Toru Imai

**Affiliations:** 1 Division of Emergency and Critical Care Medicine, Department of Acute Medicine, Nihon University School of Medicine, Tokyo 173-8610, Japan; kuwana.tsukasa@nihon-u.ac.jp (T.K.); kanai.yuma@nihon-u.ac.jp (Y.K.); yamaya.yurina@nihon-u.ac.jp (Y.Y.); takahashi.ken08@nihon-u.ac.jp (K.T.); 2 Department of Pharmacy, Nihon University Itabashi Hospital, Tokyo 173-8610, Japan; ishizuka.satoshi@nihon-u.ac.jp (S.I.); imai.toru@nihon-u.ac.jp (T.I.)

**Keywords:** augmented renal clearance, beta-lactam antibiotics, sepsis, critical care, inverse probability of treatment weighting

## Abstract

**Background/Objectives:** Sepsis is a life-threatening condition associated with high mortality. Optimal dosing strategies for β-lactam antibiotics in sepsis remain controversial, particularly in patients with renal impairment. Cefepime (CFPM) is widely used as empiric therapy; however, its appropriate initial dosing in critically ill patients is unclear. This study aimed to evaluate whether high-dose CFPM administration during the first 48 h improves survival in patients with sepsis, irrespective of renal function. **Methods:** This single-center, retrospective, observational study included adult intensive care unit (ICU) patients with sepsis who received CFPM as initial therapy between January 2017 and December 2024. Patients were categorized into High-dose (12 g within 48 h; 2 g every 8 h) and Low-dose (<12 g/48 h) groups. The primary outcome was ICU survival. To address confounding, inverse probability of treatment weighting (IPTW) based on serum creatinine was applied, with sensitivity analyses using 1% trimmed and stabilized IPTW. **Results:** Of 122 eligible patients, 84 were analyzed (High-dose: n = 27; Low-dose: n = 57). After IPTW adjustment, high-dose CFPM was significantly associated with improved ICU survival (odds ratio [OR] 5.43, 95% confidence interval [CI] 1.60–18.39, *p* = 0.0066). This association remained consistent in the 1% trimmed IPTW analysis (OR 4.07, 95% CI 1.19–13.97, *p* = 0.0256). Stabilized IPTW yielded a similar effect estimate, though without statistical significance (OR 5.43, 95% CI 0.72–41.16, *p* = 0.1017). Overall, results were consistent in direction and magnitude across models. **Conclusions:** High-dose CFPM administration during the initial 48 h was associated with improved ICU survival in patients with sepsis, independent of renal function.

## 1. Introduction

Sepsis is a life-threatening condition associated with a high mortality rate [1]. According to recent studies utilizing the updated Sepsis-3 definition, intensive care unit (ICU) mortality remains substantial, at 10.1% for sepsis without shock and 28.5% for septic shock [2]. In adults, bacterial infections are the main cause of sepsis [3]. Therefore, appropriate empirical antibiotic treatment improves survival outcomes in sepsis [4].

Traditionally, antimicrobial dosing and intervals have been adjusted based on renal function. However, during the acute phase of sepsis, augmented renal clearance (ARC), a state of enhanced renal elimination, and dilutional effects from fluid resuscitation, may lead to subtherapeutic antibiotic concentrations [5]. β-lactam antibiotics, which are widely used in sepsis, are hydrophilic and particularly affected by ARC. Cefepime (CFPM), a β-lactam antibiotic, is effective against certain Gram-positive cocci and Gram-negative rods, including *Pseudomonas aeruginosa*. Therefore, it is used as empirical therapy for sepsis, especially pneumoniae and urinary tract infection (UTI). However, the standard dosing of CFPM may result in insufficient blood concentrations. In a study of Gram-negative bacteremia in patients without renal impairment, high-dose CFPM (2 g every 8 h) was associated with reduced mortality [6].

Despite this, the optimal dosage of CFPM in patients with sepsis, particularly those with renal impairment, remains unclear. In patients with renal impairment, the standard antibiotic regimen typically involves administering a full initial dose, followed by dose reductions for subsequent doses. However, whether this regimen is effective in sepsis remains unclear. Therefore, we hypothesized that high-dose administration of CFPM during the first 48 h of treatment may improve clinical outcomes in patients with sepsis, regardless of renal function. This study aimed to investigate the association between the initial 48 h CFPM dose and patient outcomes in sepsis.

## 2. Results

### 2.1. Patient Selected Flow

**Figure 1:**
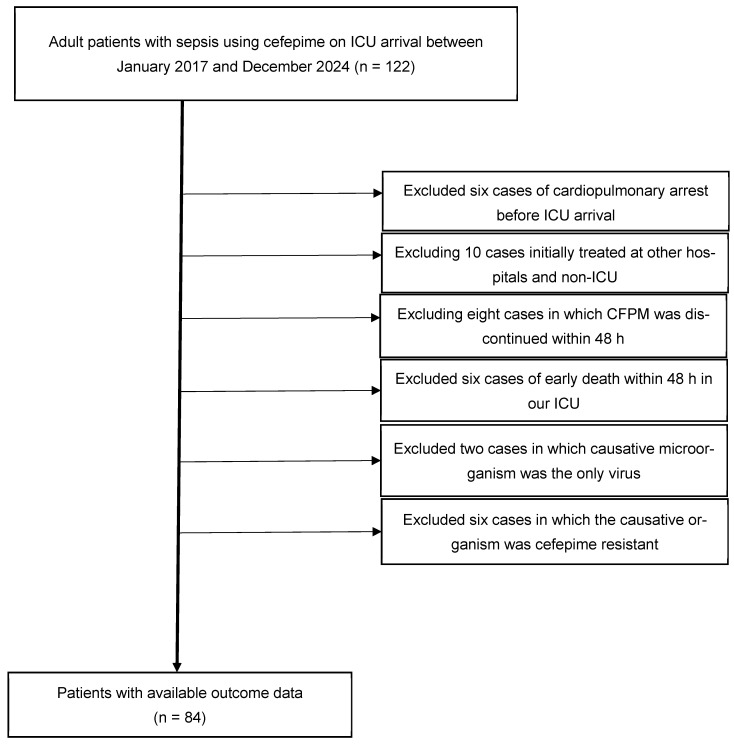
Patient selection flow. Among 122 patients screened, 38 were excluded owing to cardiopulmonary arrest before ICU arrival (n = 6), initial treatment at other hospitals or non-ICU settings (n = 10), discontinuation of CFPM within 48 h (n = 8), early death within 48 h in ICU (n = 6), viral infection only (n = 2), and CFPM-resistant pathogens (n = 6). Finally, 84 sequential cases were analyzed. Footnote: Of the six cases classified as CFPM resistant, two were pneumonia with bacteremia caused by MRSA, one was STI with bacteremia caused by MRSA, one was pneumonia caused by *Aspergillus fumigatus*, one was UTI caused by *Enterococcus raffinosus*, and one was IAI (biliary tract infection) with bacteremia caused by *Escherichia coli* (ESBL producing). Abbreviations: CFPM, cefepime; ESBL, extended-spectrum beta-lactamase; IAI, intra-abdominal infection; ICU, intensive care unit; MRSA, *Methicillin-resistant Staphylococcus aureus*; UTI, urinary tract infection.

Figure 1 illustrates the patient selection flow. Among 122 adult patients with sepsis who received CFPM upon ICU admission between January 2017 and December 2024, 38 were excluded: six with cardiopulmonary arrest before ICU arrival, 10 initially treated at other hospitals or non-ICU settings, eight in whom CFPM was discontinued within 48 h, six who died within 48 h in our ICU, two with viral infections only, and six with CFPM resistant pathogens. Finally, 84 patients were included in the analysis (High-dose group, n = 27; Low-dose group, n = 57). 

### 2.2. Baseline Characteristics

Baseline characteristics before and after inverse probability of treatment weighting (IPTW) adjustment are summarized in Table 1. Overall, 37 patients (44%) presented with septic shock, 26 (31%) had bacteremia, and the median (interquartile range [IQR]) Sequential Organ Failure Assessment (SOFA) score was 7 (5–9). The median serum Cr level was 1.22 (0.81–1.99) mg/dL. Before IPTW adjustment, the High-dose group had lower serum Cr levels (*p* < 0.0001) and lactate (*p* = 0.0127) than the Low-dose group. After IPTW adjustment, serum Cr achieved an adequate balance (absolute standardized mean difference [SMD] reduced from 0.800 to 0.089). Gram stain results were not included in the IPTW model because of polymicrobial infections and cases without identifiable pathogens.

### 2.3. Primary Outcome

**Figure 2:**
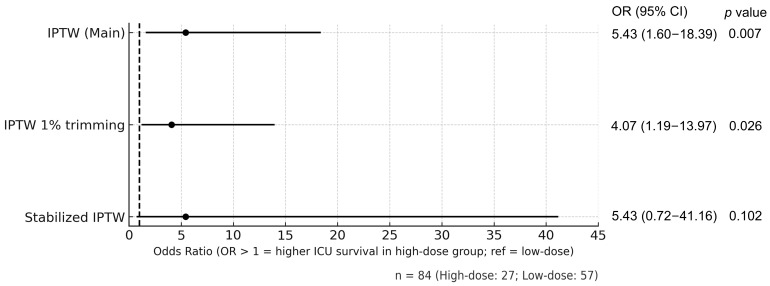
Adjusted odds ratios for ICU survival comparing high-dose versus low-dose cefepime in patients with sepsis. Adjusted ORs with 95% CIs were estimated using weighted logistic regression. Propensity scores were derived from serum creatinine and applied as IPTW. Sensitivity analyses included IPTW with 1% trimming to mitigate extreme weights and stabilized IPTW to reduce variance. OR > 1 indicates higher ICU survival in the high-dose group than in the low-dose reference group. Abbreviations: CFPM, cefepime; CI, confidence intervals; ICU, intensive care unit; IPTW, inverse probability of treatment weighting; OR, odds ratio.

Figure 2 shows the IPTW-adjusted odds ratios (ORs) for ICU survival between the High-dose and Low-dose groups. High-dose CFPM was associated with significantly improved ICU survival (OR 5.43, 95% confidence interval [CI] 1.60–18.39, *p* = 0.0066). This result remained consistent in the sensitivity analysis using 1% trimmed IPTW (OR 4.07, 95% CI 1.19–13.97, *p* = 0.0256). Although the effect estimate was similar with stabilized IPTW (OR 5.43, 95% CI 0.72–41.16, *p* = 0.1017), statistical significance was not reached, probably reflecting increased variance with stabilized weights. Overall, the findings were consistent across models in both direction and magnitude, supporting the robustness of the association.

### 2.4. Clinical Outcomes and Treatment

Clinical outcomes and treatment are presented in Table 2. After IPTW adjustment between the High-dose and Low-dose groups, ICU length of stay was 8.5 days vs. 9.8 days. The 7-day and 28-day survival rates were 98% vs. 92% and 94% vs. 61%, respectively, with missing follow-up data in 37% vs. 32% of patients. The Glasgow Coma Scale (GCS) on day 3 was 14.2 vs. 12.8, and that on day 7 was 14.4 vs. 12.8. Cumulative CFPM dose was 40 g vs. 19 g. Rates of antibiotic de-escalation were 39% vs. 49%. The minimum inhibitory concentration (MIC) of CFPM was 1.1 μg/mL vs. 1.6 μg/mL. Other treatment variables were mechanical ventilator (52% vs. 50%), hemodialysis (2% vs. 12%), low-dose steroid use (71% vs. 48%), and invasive source control (27% vs. 9%). Fluid balance variables were as follows: infusion volume in the first 24 h (2660 vs. 2401 mL), infusion volume in the first 72 h (10,166 vs. 9601 mL), urine output in the first 72 h (4832 vs. 4483 mL), and in-out balance in the first 72 h (4704 vs. 5083 mL)

## 3. Discussion

In this retrospective cohort study of critically ill patients with sepsis, high-dose CFPM administration in the first 48 h was associated with a significantly increased odds of ICU survival compared with low-dose administration after adjustment for the effects of Cr by IPTW.

In the main IPTW analysis, high-dose treatment was associated with significantly improved ICU survival. This result remained robust in the sensitivity analysis using 1% trimmed IPTW. Although the effect estimate was similar to stabilized IPTW, statistical significance was not reached, likely due to increased variance. Overall, results were consistent across models in both direction and magnitude. At the same time, the wide confidence interval observed in the stabilized IPTW analysis indicates limited precision of the effect estimate, likely attributable to the small sample size and increased variance after weighting. Therefore, the magnitude of the observed association should be interpreted with caution. A previous study of adult patients with Gram-negative bacteremia and a Cr level ≤ 1.5 mg/dL, high-dose administration of 2 g of CFPM every 8 h for at least 48 h was associated with an independent reduction in mortality [6]. Consistent with our findings, this suggests that high-dose administration of CFPM is effective, although that study excluded patients with renal impairment. Our study targeted patients with sepsis, including those with renal impairment, and demonstrated an increase in survival odds adjusted for Cr levels. This indicates that high-dose CFPM administration within the first 48 h is effective even in patients with sepsis and renal impairment. Similarly, based on evaluations of several β-lactam antibiotics, some studies have suggested that dose reduction within the first 48 h may not be advisable for antibiotics with a wide therapeutic index, such as β-lactam [7]. Patients with sepsis and acute kidney injury may have suboptimal antibiotic concentrations when the recommended dosing adjustment for kidney injury is applied during the early days of therapy [8]. Taccone et al. reported that in patients with sepsis, administering four types of beta-lactam antibiotics, including CFPM at doses adjusted for renal function, fails to maintain target blood concentrations [9]. However, no clinical studies have specifically examined the relationship between CFPM dosage and survival outcome, and our findings complement and extend previous studies.

High-dose CFPM administration during the initial 48 h may improve survival rates because CFPM blood concentrations may decrease, particularly in the early stages of sepsis. One major factor is ARC, a state of excessive renal hyperfunction that increases the renal excretion of water-soluble drugs, thereby reducing their blood concentrations [10]. ARC also occurs in sepsis; Udy AA et al. reported that ARC developed in 39.5% of sepsis cases [11]. Consequently, water-soluble β-lactam antibiotics, such as CFPM, may have lower blood concentrations than usual due to ARC. A second factor is an increased volume of distribution (Vd). Hahn RG et al. reported that a rapid infusion rate of crystalloid solutions increases Vd [12]. Furthermore, Ulldemolins M et al. noted that in critically ill patients, an increased Vd may lead to reduced blood concentrations of antibiotics on the first day of treatment, potentially resulting in inadequate therapy [13]. In this study, 44% of patients presented with septic shock, and the median fluid volume administered during the initial 72 h was 9218 mL, suggesting a likely increase in Vd. In patients with severe sepsis, a high likelihood of ARC, and large initial fluid requirements, high-dose CFPM administration during the initial 48 h may be more effective. To determine whether blood concentrations are decreasing, actual measurement of CFPM blood concentrations is necessary. However, serum CFPM concentrations were not measured in this study. In general, therapeutic drug monitoring of β-lactam antibiotics, including CFPM, is challenging to implement in daily practice owing to logistical complexity and cost. Our findings suggest that, regardless of baseline Cr levels, administering CFPM at 2 g every 8 h during the initial 48 h is beneficial, providing a strategy that can be easily incorporated into routine clinical care without the need for serum level monitoring.

In our study, pneumonia and UTI were the most common sources of infection, whereas intra-abdominal infection (IAI) was less frequent. This is because CFPM lacks antianaerobic coverage. When antianaerobic coverage is unnecessary for sepsis, CFPM has been associated with a lower 90-day mortality rate than piperacillin-tazobactam [14]. Therefore, IAI, which generally requires anaerobic coverage, has fewer sources of infection. At our institution, CFPM demonstrates high susceptibility. Based on the institutional antibiogram, the susceptibility rate of CFPM against *Pseudomonas aeruginosa* was 94% in 2024 and exceeded 90% during the study period. Based on these antimicrobial susceptibility data, infectious disease specialists at our institution recommend CFPM as empirical therapy for sepsis when anaerobic coverage is unnecessary, particularly in cases of UTI and pneumonia. In addition, the MIC of CFPM for causative bacteria was 1.1 μg/mL in the High-dose group versus 1.6 μg/mL in the Low-dose group. The clinical efficacy of β-lactam antibiotics is generally determined by the time the drug level exceeds the MIC (time above MIC: T > MIC) [15]. In a study using β-lactam antibiotics for Gram-negative bacteremia, maintaining an antibiotic concentration at least four times the MIC for 100% of the dosing interval was associated with a higher clinical cure rate [16]. Blood concentrations were not measured in this study, making T > MIC calculations impossible. However, the High-dose group likely had longer T > MIC periods than the Low-dose group. Furthermore, in studies administering CFPM at the same high dose as in this study (2 g every 8 h) for febrile neutropenia, achieving sufficient T > MIC was challenging when the MIC of the causative pathogen is > 4 μg/mL [17]. In this study, MIC data were available for only 39% of patients, which precluded meaningful and statistically stable incorporation into the propensity score model or IPTW framework. Therefore, MIC was not included as a potential effect modifier in the IPTW-based survival analysis. Further studies are needed to determine whether high-dose CFPM similarly improves survival in sepsis cases from regions and hospitals with high CFPM MICs.

A potential concern with high-dose CFPM administration is “CFPM neurotoxicity” [18]. CFPM neurotoxicity has been observed more frequently with high CFPM plasma concentrations. Ramoth et al. reported that CFPM neurotoxicity is more likely to occur with renal impairment in patients with febrile neutropenia [19]. Similarly, Jean-Michel V et al. found that CFPM encephalopathy was independently associated with chronic renal failure and CFPM blood concentrations ≥ 60 mg/dL when high-dose CFPM was administered via continuous infusion in ICU patients [20]. In our study, there were no cases where CFPM was discontinued due to neurotoxicity, although the presence of neurotoxicity among cases of septic encephalopathy cannot be completely ruled out. Although GCS is a non-specific indicator of CFPM-related neurotoxicity, GCS on day 3 and day 7 were better with the High-dose group, and no clear signal of increased CFPM-related neurotoxicity was observed. Furthermore, ARC and increased Vd may have prevented CFPM blood concentrations from reaching toxic levels even at high doses. Therefore, in clinical practice, administering high-dose CFPM during the initial 48 h and then reducing the CFPM dose according to renal impairment thereafter may reduce the risk of neurotoxicity. Importantly, CFPM-related neurotoxicity is reversible; symptoms improved in 97.5% of cases by dose reduction or discontinuation of CFPM, with a median time to improvement of 3 days [21]. Our study has demonstrated an increase in survival odds; therefore, in clinical practice, high-dose CFPM administration during the initial 48 h may be considered with careful neurological monitoring. If neurotoxicity occurs, discontinuing or reducing CFPM and switching to another antimicrobial agent is considered an appropriate management strategy.

This study had some limitations that warrant consideration. First, in our causal analysis, Cr was the only variable adjusted for, based on the directed acyclic graph (DAG) and available sample size; thus, residual confounding from unmeasured factors remains possible. Other variables, such as SOFA score and lactate, were not included in the propensity score model because they may act as mediators or colliders rather than true confounders. In particular, SOFA score includes serum creatinine, raising concerns about overadjustment. Although these variables were not included in the propensity score model, post-IPTW balance was assessed and is presented in Table 1; some residual imbalance persisted and may have influenced the estimated effect. In addition, the single-center design and relatively small sample size may limit external validity and generalizability. A randomized controlled trial would be ideal to address this, although conducting such may not be ethically feasible given the potential survival implications. Future studies could mitigate residual confounding by prospectively emulating a target trial with predefined eligibility criteria, treatment strategies, and follow-up periods. Furthermore, advanced causal inference methods, such as instrumental variable analysis or marginal structural models with time-varying exposures, may further strengthen causal interpretation when appropriate data are available. Second, although the choice between high-dose and low-dose therapy was primarily determined by Cr levels, individual physician discretion also may influence dose selection. Therefore, despite adjustment for Cr in our causal analysis, residual confounding arising from clinical judgment or other unmeasured factors cannot be fully excluded. Although IPTW improved balance for the primary confounder related to CFPM dose selection, residual imbalance remained in baseline variables such as body weight and BMI. These variables were highly correlated and were not considered major determinants of CFPM dosing in our clinical practice; however, residual confounding cannot be fully excluded. A prospective study employing a standardized protocol that allocates patients to high-dose or low-dose groups could address this concern; however, ethical considerations may limit such a design. Third, long-term outcomes, such as 90-day survival, were not evaluated in this study. Prospective studies are required to assess the impact of high-dose CFPM therapy on long-term outcomes. Fourth, although our data indicate that high-dose administration within 48 h confers a survival advantage, whether extending high-dose therapy beyond this period provides additional benefit, or conversely increases toxicity, remains unclear. Identifying the potential “turning point” between therapeutic efficacy and toxicity is an important subject for future research. Fifth, six patients who died within 48 h were excluded. Because cumulative CFPM dosage was evaluated during the initial 48 h, treatment group assignment was not feasible in these patients. Although all six patients received CFPM at treatment initiation, this exclusion may introduce survival or immortal time bias. Addressing this limitation would require modeling CFPM exposure as a time-dependent variable, such as using a Cox model with time-dependent covariates; however, this approach was incompatible with the study exposure definition, which was determined only after completion of the 48 h assessment period and could not be updated over time. Finally, CFPM was administered as a 1 h intermittent infusion; continuous or extended infusion was not performed in this study. Recent large randomized controlled trials, such as the BLING III trial, demonstrated that prolonged or continuous infusion of β-lactam antibiotics did not significantly reduce mortality compared with intermittent infusion [22]. This study cannot address whether continuous or extended administration of high-dose CFPM is beneficial. Nevertheless, our results highlight that ensuring high-dose CFPM during the initial 48 h is a key determinant of outcome, even with conventional 1 h intermittent infusion.

This study demonstrated that an early high-dose CFPM strategy may improve survival in sepsis. However, whether this strategy is applicable to other beta-lactam antibiotics remains unknown. Further investigation is required to determine the generalizability of this approach.

## 4. Materials and Methods

### 4.1. Study Design

This retrospective observational study was conducted at the Nihon University Itabashi Hospital, Tokyo. The study included consecutive adult patients admitted to the ICU with a diagnosis of sepsis between 1 January 2017, and 31 December 2024, who received CFPM as the initial antimicrobial therapy. Sepsis was defined using the Sepsis-3 criteria [23]. Patients receiving 12 g of CFPM within 48 h of admission (2 g every 8 h) were defined as the High-dose group, whereas those who received <12 g/48 h were defined as the Low-dose group. The primary outcome is ICU survival.

### 4.2. Exclusion Criteria

The exclusion criteria were cardiopulmonary arrest before ICU arrival, initial treatment with antibiotics at other hospitals and non-ICU settings, discontinuation of CFPM within 48 h, death within 48 h of ICU admission, causative microorganisms were the only virus, and infection with clearly CFPM-resistant microorganisms. Patients who underwent resuscitation for cardiopulmonary arrest prior to ICU admission were excluded because this observational study aimed to evaluate survival outcomes due to sepsis, and cardiopulmonary arrest would confound these outcomes. Patients initially treated with antibiotics at other hospitals or in non-ICU settings were also excluded, as prior antibiotic therapy may influence survival outcomes. Patients who died within 48 h of ICU admission were excluded because CFPM was discontinued within 48 h, making it impossible to evaluate survival outcomes based on CFPM dosage. Cases in which the causative microorganism was solely viral were excluded, as CFPM is not effective for viruses. Finally, patients with infections caused by clearly CFPM-resistant microorganisms were excluded, as resistance is directly associated with poor prognosis, preventing assessment of survival outcomes based on CFPM dosage, which is the objective of this study.

### 4.3. Background on CFPM Dosage Determination and Consideration of Confounding Factors Affecting ICU Survival

In our ICU, as in general ICU practices, the dosage of antibiotics is primarily determined based on the serum Cr level of the patient, reflecting renal function. Creatinine clearance, calculated from Cr and related factors, is used as a reference for dosage adjustment. For CFPM, our institutional reference dosing scheme is as follows: 2 g every 8–12 h for creatinine clearance ≥ 60 mL/min, 2 g every 12 h for 30–59 mL/min, 2 g every 24 h for 11–29 mL/min, and 1 g every 24 h for ≤10 mL/min. However, Cr and calculated creatinine clearance based on Cr are markers of chronic renal function and do not adequately capture acute changes, such as those observed in acute kidney injury (AKI) or ARC. Although Cr plays a central role in dosage decisions, clinical practice varies. For example, higher doses may be selected when ARC is suspected, whereas lower doses may be chosen owing to concern about developing AKI. The final dosage is thus ultimately determined at the discretion of the attending intensivist based on overall clinical assessment. Cr is also included in the SOFA score, which is used for sepsis diagnosis, and is associated with both organ dysfunction and patient outcomes [24]. Li et al. demonstrated that serum Cr at ICU admission was an independent predictor of 28-day mortality in adult patients with sepsis [25]. Given these factors, Cr was considered a potential confounder, as it is associated with both CFPM dosing and ICU survival.

### 4.4. Clinical Protocol

In our ICU, CFPM was administered via 1 1 h per dose as standard practice, with no extended or continuous infusion. Treatment for sepsis is generally conducted in accordance with ICU guidelines [26]. Our hospital maintains an established Antimicrobial Stewardship program overseen by the Infection Control Committee. The ICU has full-time infectious disease specialists and pharmacists onsite, which reduces instances of inappropriate antibiotic administration and supports appropriate antibiotic de-escalation. Cefepime/enmetazobactam is not approved in Japan and was therefore not used in the patients included in this study.

### 4.5. Propensity Score Adjustment

To adjust for potential confounding in the association between antimicrobial dose and survival outcomes, we employed IPTW based on propensity scores. Age and serum Cr were initially considered as candidate confounders. A causal diagram (DAG) was constructed to clarify the assumed relationships, which indicated that age functioned as an upstream variable to Cr, and Cr alone constituted a sufficient confounder. Accordingly, propensity scores were estimated using Cr alone.

The propensity score was estimated using a non-normalized logistic regression model, with treatment group assignment (High-dose or Low-dose) as the dependent variable and Cr as the independent variable. Subsequently, IPTW was calculated from the estimated propensity scores. To evaluate the association between treatment group and ICU survival, a logistic regression model weighted by IPTW was constructed, with Cr included as a covariate. Covariate balance before and after weighting was assessed using SMDs to confirm the adequacy of adjustment. Sensitivity analyses were performed as follows: (1) IPTW with 1% weight trimming and stabilized IPTW to assess robustness; (2) analysis restricted to cases treated with CFPM alone, excluding those who received concomitant antibiotics, categorized into the High-dose and Low-dose groups, with ICU survival compared using IPTW analysis with Cr as a confounding factor.

### 4.6. Sample Collection and Measurement

The following data were collected from clinical records: Baseline characteristics: age, sex, body mass index, weight; medical history: chronic kidney disease, diabetes and steroid use; patient profile: septic shock, bacteremia, SOFA score (assessing organ dysfunction across six organ systems) [24], and blood sampling data including Cr; infectious source: pneumonia, UTI, soft tissue infection, IAI, infectious endocarditis, and unknown; causative bacteria: Gram stain classification of causative bacteria—Gram-negative rod, Gram-positive cocci, and others (oral commensal bacteria, Gram-negative cocci, Gram-positive rods, and fungus; antibiotics: CFPM dose in first 48 h, concomitant antibiotic use; clinical outcomes: length of ICU stay, 7-day survival, 28-day survival, time from sepsis diagnosis to CFPM initiation, cumulative CFPM dose, duration of CFPM administration, duration of total antibiotics, and de-escalation; microbiological data: MIC of CFPM against the causative bacteria; treatment variables: ventilator use, hemodialysis, low-dose steroids, and invasive source control; and clinical assessments and fluid balance: GCS on days 3 and 7, total infusion volume in first 24 and 72 h, total urine output in first 72 h, and total in-out balance in first 72 h.

### 4.7. Statistical Analysis

Statistical analyses were conducted using JMP version 13.0.0 (SAS Institute Inc., Cary, NC, USA) and Python version 3.10.13 (Python Software Foundation, Wilmington, DE, USA). The normality of continuous variables was assessed, and non-normally distributed variables were identified. Continuous variables were compared between the High-dose and Low-dose groups using the Mann–Whitney U test. Categorical variables were compared using Fisher’s exact or Pearson’s chi-square test, as appropriate. Statistical significance was defined as a two-sided *p*-value < 0.05. Data are presented as medians and IQRs due to non-normal distributions. Propensity score estimation and IPTW were performed using the statsmodels package (version 0.13.5) in Python. Propensity scores were estimated using logistic regression (statsmodels.Logit) with a single covariate (Cr). IPTW weights were calculated using the standard (unstabilized) method. Sensitivity analyses included 1% weight trimming and stabilized IPTW. Weighted logistic regression models (statsmodels.GLM) were used to estimate the association between treatment group and survival. These procedures were designed to approximate standard IPTW methodology as implemented in the WeightIt and survey packages in R (version 4.3.2; R Foundation for Statistical Computing, Vienna, Austria), without incorporating complex survey design elements.

## 5. Conclusions

In critically ill patients with sepsis, high-dose CFPM administration (2 g every 8 h, total 12 g in 48 h) was associated with higher ICU survival odds compared with low-dose administration after adjustment for Cr. Our findings suggest that administering high-dose CFPM during the initial 48 h may improve survival regardless of renal function.

## Figures and Tables

**Table 1:** Baseline characteristics of patients in the High-dose and Low-dose groups.

	Overall (n = 84)	Before IPTW Adjustment			After IPTW Adjustment		SMD Before	SMD After
		High-Dose Group (n = 27)	Low-Dose Group (n = 57)	*p* Value	High-Dose Group	Low-Dose Group		
Age (years)	79 (68–86)	77 (61–81)	79 (72–87)	0.0623	68.4	79.0	0.503	0.777
Male	53 (63%)	17 (63%)	36 (63%)	0.9862	63%	62%	0.004	0.017
BMI (kg/m^2^)	20 (17–23)	20 (17–24)	20 (17–22)	0.7123	22.2	19.64	0.111	0.564
Weight, kg	50 (42–60)	54 (42–65)	50 (42–58)	0.4521	60.1	50.0	0.214	0.677
Medical history								
CKD	11 (13%)	4 (15%)	7 (12%)	0.7478	9%	11%	0.073	0.057
Diabetes	26 (31%)	8 (30%)	18 (32%)	0.8568	25%	30%	0.042	0.118
Steroid use	12 (14%)	6 (20%)	6 (11%)	0.1525	15%	11%	0.315	0.105
Patient Profile								
Septic shock	37 (44%)	9 (33%)	28 (49%)	0.1734	52%	49%	0.321	0.059
Bacteremia	26 (31%)	5 (19%)	21 (37%)	0.0898	43%	34%	0.413	0.169
SOFA score	7 (5–9)	7 (4.5–8.5)	8 (6–10)	0.0929	7.7	7.7	0.408	0.018
Lactate (mmol/L)	3.0 (1.6–5.3)	2.1 (1.4–4.0)	4.5 (2.0–5.7)	0.0127	3.6	4.3	0.597	0.247
Cr (mg/dL)	1.22 (0.81–1.99)	0.84 (0.61–1.05)	1.52 (1.04–2.22)	<0.0001	1.59	1.71	0.800	0.089
WBC (10^4^/µL)	11 (7–18)	10 (8–15)	11 (7–18)	0.9618	21.3	13.3	0.056	0.513
N/L ratio	14 (5–26)	13 (5–31)	14 (7–25)	0.5056	27.6	27.6	0.037	0.001
CRP (mg/dL)	10 (2–19)	10 (2–17)	10 (2–20)	0.8744	16.0	11.1	0.043	0.422
Infectious source								
Pneumonia	52 (62%)	20 (43%)	32 (56%)	0.1139	70%	61%	0.378	0.184
UTI	23 (27%)	6 (18%)	17 (30%)	0.4655	28%	27%	0.172	0.02
STI	2 (2%)	0 (0%)	2 (3%)	0.3246	0%	3%	0.267	0.234
IAI	2 (2%)	1 (4%)	1 (2%)	0.5842	2%	2%	0.118	0.041
IE	1 (1%)	0 (0%)	1 (2%)	0.4887	0%	1%	0.187	0.164
unknown	4 (5%)	0 (0%)	4 (7%)	0.1584	0%	8%	0.435	0.404
Gram Stain ^1^								
GNR	43	13	30	0.7010	-	-	-	-
GPC	29	7	22	0.2540	-	-	-	-
Others ^2^	15	6	9	0.4722	-	-	-	-
Antibiotics								
CFPM dose in the first 48 h (g) ^3^	8(8–12)	12 (12–12)	8 (4–8)	<0.0001	-	-	-	-
Concomitant antibiotic use with CFPM	25 (30%)	11 (29%)	15 (26%)	0.1817	43%	26%	0.305	0.371

^1^ Gram staining classified bacteria identified as causative agents from cultures obtained from the infection site (such as sputum culture for pneumonia) and from blood cultures. All patients underwent microbiological culture. Multiple bacterial infections occurred in three cases in the High-dose group and 12 in the Low-dose group. No bacteria were detected in four cases in the High-dose group and eight in the Low-dose group. Gram stain results were not included in the IPTW model owing to the presence of polymicrobial infections and cases without identifiable pathogens. ^2^ Other bacteria were as follows: in the High-dose group, six cases were oral commensal bacteria; in the Low-dose group, five cases were oral commensal bacteria, one case was Gram-negative cocci (GNC), and three cases were Gram-positive rods (GPR). ^3^ Since the CFPM dose in the first 48 h (g) was used as the exposure-defining variable, it was not included in the covariate balance assessment. Data are expressed as median (interquartile range, IQR). *p*-values are shown for descriptive purposes only. Covariate balance was assessed based on standardized mean differences (SMD), which are independent of sample size. Abbreviation: BMI, body mass index; CFPM, cefepime; CKD, chronic kidney disease; Cr, creatinine; CRP, C-reactive protein; GNR, Gram negative rod; GPC, Gram positive cocci; C-reactive protein; IAI, Intra–abdominal infection; IE, infectious endocarditis; N/L ratio, Neutrophil/Lymphocyte ratio; SMD, standardized mean difference; SOFA, sequential organ failure assessment; STI, soft tissue infection; UTI, urinary tract infection; WBC, white blood cell.

**Table 2:** Clinical outcomes and treatment of patients in the High-dose and Low-dose groups.

	Overall (n = 84)	Before IPTW Adjustment			After IPTW Adjustment	
		High-Dose Group (n = 27)	Low-Dose Group (n = 57)	*p* Value	High-Dose Group	Low-Dose Group
Outcomes						
ICU stay (day)	8 (5–12)	8 (5–11)	8 (4–13)	0.8703	8.5	9.8
7-day survival	78 (93%)	26 (96%)	52 (91%)	0.4215	98%	92%
28-day survival Missing, n (%) ^a^	39 (68%) 28 (33%)	15 (88%) 10 (37%)	24 (62%) 18 (32%)	0.0585	94%	61%
Antibiotic						
Time from sepsis diagnosis to CFPM initiation (min)	84 (60–145)	98 (70–174)	79 (56–124)	0.0748	112.3	127.3
Cumulative CFPM dose (g)	22 (12–30)	30 (24–42)	14 (8–24)	<0.0001	40	19
Duration of CFPM (day)	5 (3–7)	5 (4–7)	5 (3–7)	0.1932	6.7	5.4
Duration of total antibiotics (days)	8 (7–10)	8 (7–9.5)	8 (7–10)	0.7189	11.3	9.2
De-escalation	42 (50%)	14 (52%)	28 (49%)	0.8153	39%	49%
MIC of CFPM (μg/mL) Measured, n (%)	1 (1–1) 33 (39%)	1 (1–1) 9 (33%)	1 (1–1) 24 (42%)	0.4705	1.1	1.6
Treatment						
Ventilator	41 (49%)	13 (48%)	28 (49%)	0.9335	52%	50%
Duration of mechanical ventilation (day)	2 (0–6)	2 (0–5.5)	2 (0–7)	0.9678	5.2	5.2
Hemodialysis	10 (12%)	1 (4%)	9 (16%)	0.1102	2%	12%
Low-dose steroid	41 (49%)	14 (52%)	27 (47%)	0.7010	71%	48%
Invasive source control	8 (10%)	2 (7%)	6 (11%)	0.6493	27%	9%
Clinical outcome						
GCS day 3 Missing, n (%) ^b^	14 (12–15) 2 (2%)	14 (14–15)	14 (12–15) 2 (4%)	0.0168	14.2	12.8
GCS day 7 Missing, n (%) ^c^	14 (13–15) 17 (20%)	15 (14–15) 8 (30%)	14 (13–15) 9 (16%)	0.0235	14.4	12.8
Total infusion volume in first 24 h, mL	2240 (1847–2892)	2391(2073–2948)	2170 (1830–2810)	0.3834	2660	2401
Total infusion volume in first 72 h, mL Missing, n (%)	9218 (7493–11,574) 1 (1%)	9643 (7503–11,362)	9043 (7513–11,734) 1 (2%)	0.7855	10,166	9601
Total urine output in first 72 h, mL Missing, n (%)	4465 (2650–6236) 1 (1%)	5230 (3249–7593)	4274 (2599–5855) 1 (2%)	0.1176	4832	4483
Total in-out balance in first 72 h, mL Missing, n (%)	4269 (2567–6975) 1 (1%)	3352 (2047–6393)	4449 (2820–7160) 1 (2%)	0.1767	4704	5083

Data are expressed as median (interquartile range). ^a^. Twenty-eight-day survival data were unavailable for 10 patients in the High-dose and for 18 patients in the Low-dose groups, respectively, due to loss to follow-up after hospital discharge. ^b^. One patient died before day 3, and one was discharged from the ICU on day 2. ^c^. Seven patients were lost to follow-up due to early ICU discharge, and one due to early death in the High-dose group; six cases were lost to follow-up due to early ICU discharge, and three due to early death in the Low-dose group. CFPM, cefepime; GCS, Glasgow Coma Scale; ICU, intensive care unit; MIC, Minimum Inhibitory Concentration.

## Data Availability

The datasets generated and/or analyzed in this study have been fully anonymized to ensure patient privacy protection. Due to institutional and ethical restrictions, these data are not publicly available. However, the anonymized dataset (including the Excel file used for the analysis) is available from the corresponding author upon reasonable request and subject to approval by the institutional review board.

## Abbreviations

The following abbreviations are used in this manuscript:

BMI	body mass index
CFPM	cefepime
Cr	creatinine
ICU	intensive care unit
IPTW	inverse probability of treatment weighting
DAG	directed acyclic graph
SOFA	sequential organ failure assessment
UTI	urinary tract infection
IAI	intra-abdominal infection
MIC	minimum inhibitory concentration
GCS	Glasgow coma scale
ARC	augmented renal clearance
AKI	acute kidney injury
